# Targeted delivery of doxorubicin and therapeutic FOXM1 aptamer to tumor cells using gold nanoparticles modified with AS1411 and ATP aptamers

**DOI:** 10.22038/IJBMS.2023.71129.15452

**Published:** 2023

**Authors:** Aref Abdollahzade, Hoda Rahimi, Elnaz Yaghoobi, Mohammad Ramezani, Mona Alibolandi, Khalil Abnous, Seyed Mohammad Taghdisi

**Affiliations:** 1 School of Pharmacy, Mashhad University of Medical Sciences, Mashhad, Iran; 2 Pharmaceutical Research Center, Pharmaceutical Technology Institute, Mashhad University of Medical Sciences, Mashhad, Iran; 3 Department of Chemistry and Biomolecular Sciences, University of Ottawa, 10 Marie-Curie, Ottawa, ON K1N 6N5, Canada; 4 Department of Medicinal Chemistry, School of Pharmacy, Mashhad University of Medical Sciences, Mashhad, Iran; 5 Targeted Drug Delivery Research Center, Pharmaceutical Technology Institute, Mashhad University of Medical Sciences, Mashhad, Iran; 6 Department of Pharmaceutical Biotechnology, School of Pharmacy, Mashhad University of Medical Sciences, Mashhad, Iran

**Keywords:** Aptamers, Antineoplastic agents, Cell survival, Doxorubicin, Metal nanoparticles, Nucleolin

## Abstract

**Objective(s):**

A targeted delivery platform was prepared to co-deliver both doxorubicin (Dox) as an anticancer drug and FOXM1 aptamer as a therapeutic substance to breast cancer cells (4T1 and MCF-7) to reduce Dox side effects and increase its therapeutic efficacy. The targeted system (AuNPs-AFPA) consisted of FOXM1 aptamer, AS1411 aptamer (targeting oligonucleotide), ATP aptamer, and gold nanoparticles (AuNPs) as a carrier.

**Materials and Methods:**

AuNPs were synthesized by reduction of HAuCl4. Next, after pegylation of ATP aptamer, FOXM1 aptamer-PEGylated ATP aptamer conjugate (FPA) was prepared. Then, the AS1411 aptamer and FPA were exposed to the AuNPs surface through their thiol groups. Subsequently, Dox was loaded into the complex to form a targeted therapeutic complex.

**Results:**

The data of the MTT assay displayed that the targeted complex could remarkably reduce cell viability rate in target cells due to the overexpression of nucleolin on their cell membranes compared to nontarget cells, showing the targeting ability of AuNPs-AFPA-Dox. The in vivo antitumor effect confirmed that AuNPs-AFPA-Dox was capable of remarkably diminishing tumor growth relative to the free Dox in mice bearing 4T1 tumor cells.

**Conclusion:**

The results confirmed that the targeted system improved the therapeutic effect by loading high amounts of Dox alongside the presence of the therapeutic effect of FOXM1 aptamer. Finally, it can be concluded that AuNPs-AFPA-Dox by enhancing antitumor effectiveness and reducing toxicity toward non-target cells, can be used potentially as an effective strategy for the treatment of breast cancer.

## Introduction

Breast cancer is one of the most common cancers in women, and it is categorized among the main leading causes of cancer death worldwide (1, 2). Despite many advances in the initial treatment of breast cancer, like radiotherapy, surgical procedures, chemotherapy, and hormone therapy, the death rate of breast cancer is still growing (3). Chemotherapy is one of the main options for cancer treatment. However, it causes intense adverse effects on normal tissues because of its lack of selection between cancer cells and normal cells in the body (4). Based on reports, during chemotherapy, the amount of administrated doses of chemotherapy drugs entering normal cells is around 90%, which causes severe complications in the patients (5, 6). Doxorubicin (Dox), an anthracyclines antitumor drug, is used to treat a variety of cancers, especially breast cancer. However, due to its short half-life and especially cumulative dose-dependent cardiac toxicity, its clinical use has been limited (7, 8). Therefore, to reduce unwanted side effects and increase the delivery of drugs to the target cancer cells, the design of new targeted drug delivery systems by applying nanocarriers and oligonucleotide therapeutic agents is necessary (9-11).

AuNPs have been widely used and studied in the targeted delivery of therapeutic materials because of their unique characteristics such as small size, high surface-to-volume ratio, simple surface modification with drugs and targeting agents, high stability, high dispersion, biocompatibility, and their accumulation in tumor tissues. Also, due to their ease of synthesis and their low toxicity *in vivo*, recently AuNPs have been considered drug delivery carriers for the diagnosis and treatment of diseases such as cancer (12). In general, the surface of AuNPs is conjugated with drugs or biological molecules to bind to their target at the tumor site (13). 

Today, one of the new strategies for the treatment of cancerous tissues is targeting drug delivery systems using aptamers as potential targeting agents (14, 15). Aptamers are single-stranded DNA or RNA (16, 17). Aptamers can be attached to their corresponding targets with high specificity and sensitivity (18). Because of the three-dimensional structures of aptamers and their characteristics, aptamers have highlighted advantages over antibodies and other biological targeting agents, such as a high trend to attach to target molecules, high thermal stability, low cost, ease of chemical modification and production, cost and time-effective synthesis, non-toxicity, and having a smaller size than antibodies which allows them to penetrate better to solid tumors (19, 20). Unlike monoclonal antibodies and peptides, aptamer-based DNA origami do not stimulate the immune response, which makes them further useful in the long treatment and also makes them good nominees for targeted drug delivery (21, 22). 

The AS1411 aptamer (AS1411) is a 26-mer guanine-rich single-stranded DNA that can form a stable structure against nuclease degradation in serum (23). This aptamer can bind with high sensitivity and specificity to nucleolin. Nucleolin or C23 is a cell-surface protein in the nucleus of normal cells that overexpresses on membranes of tumor cells such as prostate, breast, lung, and stomach cancers (24). The function of nucleolin is to play a role in the transport of accompanying molecules from the membranes of cells to their nucleus, DNA replication, and cell proliferation (25). AS1411 by bounding to nucleolin can transport itself and its accompanying drug system to the cytoplasm and nucleus of tumor cells (18, 26). Also, this aptamer in high concentrations can lower the proliferation of tumor cells through a regulatory effect on the activity of helicase in DNA division (18, 27).

FOXM1 aptamer (FOXM1 Apt) is a 42-mer single-stranded DNA that is bonded to the Forkhead Box Protein M1 (FOXM1). FOXM1 is an effective transcription factor in the cell proliferation cycle through its binding with target gene promoters, which are mainly located in the cell nucleus (28). Abnormal overexpression of FOXM1 is associated with cell proliferation, metastasis, and tumorigenesis (29). In most human cancers especially in breast cancer, FOXM1 protein is overexpressed, which can cause resistance to chemotherapy. Studies have shown that by binding FOXM1 Apt to its target and suppressing the FOXM1 protein, tumor cells can be vulnerable to death, and the growth of cancer cells is reduced (18, 19). By binding of FOXM1 protein to FOXM1 Apt, the function of FOXM1 protein is disrupted and its transcriptional activities suppressed (19). In general, studies on FOXM1 Apt offer new insights into the treatment of cancers and other diseases (23).

ATP aptamer (ATP Apt), (adenosine-5’-triphosphate) is a 25-base single-stranded oligodeoxynucleotide that has a high affinity for ATP unlike other triphosphate nucleotides (30). Because of the considerable difference in the concentration of ATP inside and outside the cells, the use of ATP Apt can be an effective way to regulate the rate of drug delivery into the target cell (31). Studies have shown that ATP Apt in the targeted drug delivery system can be used as a stimulant for the controlled release of anticancer drugs, and also it can selectively release Dox in an ATP-rich environment (32). In this study, to prepare a DNA nanostructure on the surface of AuNPs, the end of ATP Apt needs to be pegylated by ester bonding with PEG-COOH. Pegylation improves the performance of the targeted drug delivery system by protecting it against degradation by metabolic enzymes (27, 33).

**Scheme 1:**
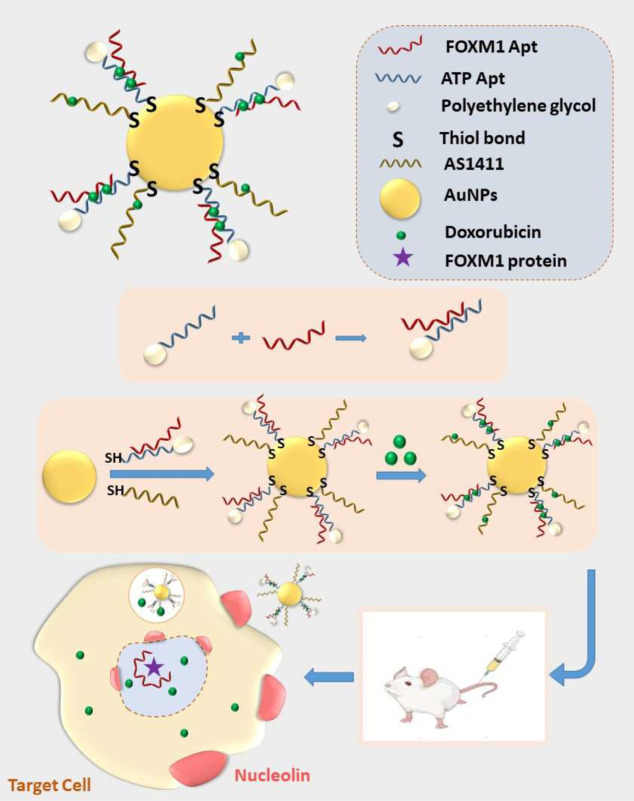
Schematic illustration of AuNPs-AFPA-Dox and its function in target breast cancer cells (4T1) *in vivo*

In a recent study, a new approach in the treatment of breast cancer was reported by preparing a novel Dox-DNA nanostructure on the surface of AuNPs that is targeted by aptamers for selective transfer of Dox to tumor cells (Scheme 1). There are three kinds of aptamers in this structure, containing FOXM1 Apt as a therapeutic agent, AS1411 as a targeting element, and ATP Apt as a controlled release agent of anticancer drugs. The presence of FOXM1 Apt as a therapeutic aptamer and Dox as a chemotherapeutic drug together in the design of the delivery platform increases the efficiency of cancer therapy and decreases toxicity for nontarget cells compared to free drugs by decreasing the required amount of Dox. *In vivo* and *in vitro* studies were investigated to assess the therapeutic efficacy of the designed targeted system.

## Materials and Methods

***Materials***

All the ssDNA sequences were purchased from Microsynth AG (Switzerland) (Table S1). Doxorubicin hydrochloride (Dox), N-Hydroxysuccinimide (NHS), dimethyl sulfoxide (DMSO), and N-(3-Dimethylaminopropyl)-N′-ethylcarbodiimide hydrochloride (EDC) were procured from Sigma-Aldrich (USA). RPMI 1640 medium, fetal bovine serum (FBS), and penicillin-streptomycin were obtained from Gibco (Germany). Carboxylic acid poly (ethylene glycol) (PEG) (5 kDa) was supplied by NANOCS (USA). 3K centrifugal devices were ordered from PALL (USA). 

***Cell lines***

CHO (Chinese hamster ovary cell), MCF-7 (human breast cancer cell), and 4T1 (mouse breast cancer cell) cell lines were supplied by the Pasteur Institute of Iran. All cells were incubated in RPMI 1640 supplemented with 10% FBS and 1% penicillin-streptomycin.

***Synthesis of AuNPs***

**AuNPs were prepared by the reduction of HAuCl**_4_ by citrate according to a published protocol (34). Briefly, after boiling and stirring vigorously 100 ml HAuCl_4_ (1 mM) and 10 ml sodium citrate (38.8 mM) solution were combined. It led to a clear color alteration from light yellow to wine red. Following boiling for 15 min, the container containing the sample was left at room temperature. After centrifuging the prepared AuNPs sample at 15,000 × g for 20 min at 4 °C, the supernatant was discarded and the AuNPs were resuspended again in ultrapure water (35). Using an extinction coefficient of 2.7 × 10^8^ M^-1^ cm^-1^ at λ = 520 nm for AuNPs (15 nm), the concentration of required AuNPs was calculated. Characteristics of the prepared AuNPs, including zeta potential, morphology, and size were assessed by a particle size analyzer (Malvern, UK) and transmission electron microscopy (TEM) (CM120, Philips, Holland).

***Preparation of PEGylated ATP aptamer***

Five microliter carboxylic acid PEG (3.5 mM) was incubated with 10 μl NHS (175 mM) and 10 μl EDC (350 mM) with gentle stirring for 20 min. Then, 40 µl ATP Apt was transferred to the mixture to make the final concentration of ATP Apt equal to 70 µM and incubated for 3 hr at room temperature. Next, the sample was centrifuged (6860×g for 5 min) using a 3K centrifugal device to remove excess EDC, NHS, and unbound PEG. The formation of PEGylated ATP Apt (PA) was analyzed and confirmed using 2.5% agarose gel electrophoresis (36).

***Preparation of the FOXM1 Apt and PEGylated ATP Apt conjugate***

To bind FOXM1 Apt to its supplemented aptamer, PA, different ratios of them (1:1, 1:2, and 1:4 ratios) were incubated in a 1:1 mixture of phosphate buffer saline (pH 7.4, 10 mM PBS) and deionized water for 3 hr. Finally, the formation of FOXM1 Apt and PA conjugate (FPA) was analyzed using 2.5% agarose gel electrophoresis.

***Preparation of Apt-modified AuNPs***

The amount of AuNPs was measured to be 7 nM. The ratio of AS1411 to FPA was 1:1 to prepare the AuNPs-AFPA. TCEP is required to bind AS1411 and FPA to AuNPs because these two aptamers have a thiol group. To prepare 150 µl of the AuNPs-AFPA (final concentration 0.33 µM), 2 µl AS1411 (25 µM) and 3 µl TCEP (5 µM) were incubated in a microtube and also 12.5 µl PA (4 µM) and FOXM1 Apt (1 µM) conjugate and 18.75 µl TCEP (5 µM) were incubated in another microtube for 1 hr. The contents of both microtubes were mixed with 99 µl AuNPs (7 nM) and 14.75 µl citrate buffer and left overnight at room temperature. Finally, to demonstrate the preparation of the AuNPs-AFPA, 2.5% agarose gel was utilized.

Particle size, dispersion, and surface charge (zeta potential, Mv) were calculated using a Zeta Sizer by dynamic light scattering (DLS) (NANO-ZS, Malvern, UK). The transmission electron microscope (TEM, Tecnai F20, 200 kV) was used to obtain the image of AuNPs (37).

***Stability test by using NaCl solution***

Into every 4 separated microtubes, 100 µl AuNPs (7 nM) and into the other 4 separated microtubes, 100 µl of AuNPs-AFPA (final AuNPs concentration was 7 nM) were added. Then, different volumes of 1 M NaCl solution (0, 3, 6, and 10 µl, respectively) were transferred to each of the microtubes. After 5 min, the spectra absorbance of all samples was measured by a microplate reader (450–700 nm).

***Serum stability of AuNPs-AFPA***

After incubation of AuNPs-AFPA (12 μM) with fresh human serum at 37 °C for 7 hr, phenol-chloroform was applied to isolate the DNA structure and it was centrifuged at 4 °C (9500×g for 5 min). Then, the pellet-containing complex was dissolved by adding deionized water. Finally, to analyze the serum stability of the complex, it was run on a 2.5% agarose gel electrophoresis.

***Dox loading onto the Apt-modified AuNPs***

Growing amounts of AuNPs-AFPA (0–300 nM) were added to a constant amount of Dox (1 µM) in 10 mM PBS and incubated for 1 hr to analyze Dox loaded onto the AuNPs-AFPA. To evaluate the fluorescence spectra of Dox, a synergy H4 microplate was deployed (λ_EM_ = 500-700 nm and λ_Ex_ = 480 nm) (BioTek, USA).

***Dox release profile***

To measure pH-triggered Dox release from AuNPs-AFPA-Dox (containing 1 µM Dox), AuNPs-AFPA-Dox was individually incubated in citrate buffer with pH 5.5 (simulation of intracellular conditions) and PBS with pH = 7.4 (simulation of blood conditions) in a shaker incubator for 72 hours at 37 °C and 100 rpm. Then, the released Dox was separated during different time intervals (0–72 hr) by centrifuging using a 3K filter (5 min at 10,000×g). Finally, the Dox quantity was calculated by measuring the fluorescence spectra of Dox (λ_EM_ = 500–700 nm and λ_Ex_ = 480 nm).

***Cell viability assay***

To check cell viability, the IC_50_ values of Dox were determined using the dose-escalation study of Dox for CHO, 4T1, and MCF-7 cell lines (0.2, 0.15, and 0.3 µM, respectively). 

Cell lines were seeded in 96-well plates (5 × 10^5^ cells/well) at 37 °C. After 20 hr, the cells were incubated with the following groups based on the result of IC_50_ for 3 hr (5 replicates were considered for each concentration). Next, the culture medium containing Dox was eliminated and replaced with a fresh culture medium. Subsequently, following 72 hr of incubation, 20 µl of MTT solution (5 mg/ml in PBS) was mixed in each well and left for 3 hr. Then, after aspiration MTT, 100 µl DMSO was added to each well and stirred on a shaker at 110 rpm for 10 min. Eventually, the absorbance of each well was recorded by a microplate reader (A_570_ and A_630_).

The groups used are as follows:

1) Dox-DNA nanostructure including AuNPs, AS1411, FOXM1 Apt, PEGylated ATP Apt, and Dox (AuNPs-AFPA-Dox)

2) DNA nanostructure including AuNPs, AS1411, FOXM1 Apt, PEGylated ATP Apt (AuNPs-AFPA)

3) Dox-DNA nanostructure including AuNPs, FOXM1 Apt, PEGylated ATP Apt, and Dox (AuNPs-FPA-Dox)

4) Dox-DNA nanostructure including AuNPs, AS1411, PEGylated ATP Apt, and Dox (AuNPs-APA-Dox)

5) Dox-DNA nanostructure including AuNPs, AS1411, and Dox (AuNPs-A-Dox)

6) Drug-free gold nanoparticles (AuNPs)

7) Dox

***Cellular uptake***

Flow cytometry analysis was conducted to evaluate cellular internalization. MCF-7, CHO, and 4T1 cells were seeded in a 6-well plate (2×10^5^ cells/well). After 24 hr incubation, cultured cells were treated with AuNPs-AFPA-Dox, AuNPs-FPA-Dox (final Dox amount was 3 µM), and Dox (3 µM) for 3 hr in an incubator. Next, the supernatant of each well was replaced with a fresh culture medium. Following 2 hr re-incubation, the cells were rinsed and trypsinized, and the fresh culture medium was added to neutralize trypsin. After centrifuging the cells at 1500 rpm for 5 min, a cell suspension was produced by transferring 10 mM PBS. Ultimately, to evaluate cellular internalization, Dox fluorescence was analyzed using flow cytometry (BD Biosciences, USA). The obtained data were applied in FlowJo 7.6.1 software.

***Fluorescence imaging***

After 24 hr of culturing MCF-7, CHO, and 4T1 cell lines in the 96-well plate (5 × 10^4^ cells/well), the cells were treated with AuNPs-AFPA-Dox (final drug concentration was 3 µM) and 3 µM Dox (2 replicates were considered for each condition) for 3 hr at 37 °C. Subsequently, 2 hr later, the cellular internalization of the nanoparticle and drug into all 3 cell lines was investigated using an inverted fluorescence microscope (CETI, UK).

***In vivo study***

All animal experiments were conducted under the institutional ethical committee and research advisory committee of Mashhad University of Medical Sciences (IR.MUMS.PHARMACY.REC.1399.005). To induce cancer in BALB/c mice, 4T1 cells (3 × 10^5^ cells) were injected into the right flank of each mouse weighing about 15–20 g. After the tumor size was approximately 50 mm^3^, all BALB/c mice (female, 4–6 weeks old) were classified into 5 various categories (n = 4). The treatment including AuNPs-AFPA-Dox, AuNPs-AFPA, AuNPs-FPA-Dox, PBS, and Dox as free drug (Dox equivalent concentration 0.15 mg/Kg) was injected as a single dose into mice via the tail vein. The tumor size was calculated every 3 days for 30 days by deploying a caliper and using this formula: width × length × height × 0.5. Also, within 30 days after injection, the survival and weight in mice were monitored to evaluate their systemic toxicity. 

***EX vivo*** **imaging**

After 6 hr post-injection of AuNPs-AFPA-Dox and Dox (Dox amount 0.15 mg/Kg) into female BALB/c mice bearing 4T1 tumor cells with size nearly 200 mm^3^ via tail vein, mice were euthanized and their organs such as liver, kidney, lung, heart, tumor, and spleen were dissected and rinsed with normal saline. Then, the biodistribution of Dox and complex in each organ were evaluated by the animal fluorescence imaging device (KODAK IS *in vivo* imaging system) at λ_EX_ = 480 nm and λ_EM_ = 550 nm and compared with each other.

***Statistical analysis***

The data are Means±SD (standard deviation), (n = 4). In this study, One-way ANOVA, Two-way ANOVA, and Post-test Tukey-Kramer were considered for comparison among different groups and control. The data were considered statistically significant at *P*˂0.05.

## Results

***Characterization of the synthesized AuNPs and AuNPs-AFPA***

**Figure 1:**
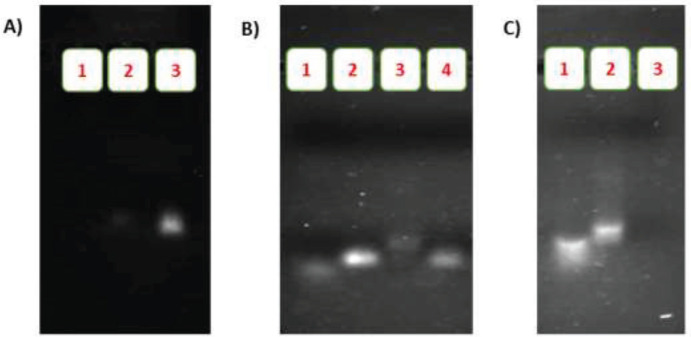
**(**A) Confirmation of the formation of the PA by using agarose gel electrophoresis: (1) Solution under the 3K filter, (2) Solution on the 3K filter, (3) ATP Apt. (B) Confirmation of the formation of the FPA by using agarose gel electrophoresis: (1) PA, (2, 4) FOXM1 Apt, (3) FPA. (C) Confirmation of the formation of the AuNPs-AFPA by using agarose gel electrophoresis: (1) AS1411, (2) FPA, and (3) AuNPs-AFPA

The formation of PA, FPA, and AuNPs-AFPA was analyzed and confirmed using agarose gel electrophoresis. As displayed in Figure 1A, the band of PA (lane 2) almost disappeared on the gel because of lower access of GelRed to the PEGylated aptamer relative to the band of the control sample (lane 3), verifying the formation of the PEGylated structure. However, before being pegylated, there was a sharp band for ATP Apt (lane 3).

To bind FOXM1 Apt to its complementary aptamer, PA, different ratios of them (1:1, 1:2 (Figure S1), and 1:4 ratios, respectively) were incubated for 3 hr. After incubation the mole ratio 1:4 of FOXM1 Apt:PA for 3 hr, electrophoresis on 2.5% agarose gel was used to assess their binding. As shown in Figure 1B, the migration of the band of FPA (lane 3) was retarded compared to bands of control samples (lanes 1 and 2), confirming the formation of the FPA with the mole ratio 1:4 of FOXM1 Apt:PA.

As shown in Figure 1C, after binding aptamers to AuNPs, the absence of a band which was corresponding to this mixture (lane 3) compared to bands of control samples (lanes 1 and 2), indicates the formation of the AuNPs-AFPA structure completely and confirms the connection of AS1411 and FPA to AuNPs in the mole ratio of 1:1.

Moreover, the hydrodynamic sizes of AuNPs and AuNPs-AFPA were 11.99 nm and 26.07 nm, respectively, as measured by DLS (Table S2). The reason for increased particle size in AuNPs-AFPA was the binding of AuNPs to aptamers. The AuNPs scattering index (PDI) showed that all AuNPs had a uniform size distribution (less than 0.3). However, the PDI for AuNPs-AFPA increased due to different folding of aptamers and their flexibility. The Zeta potential for AuNPs and AuNPs-AFPA were -25.9 mV and -10.3 mV, respectively. The variation among zeta potential results is related to aptamers that can cover the surface of AuNPs.

**Figure 2:**
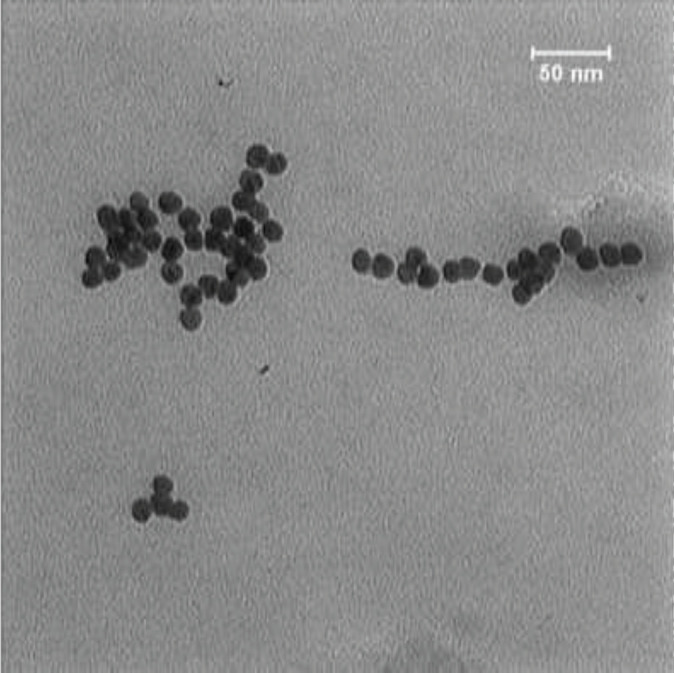
TEM image of AuNPs

As shown in Figure 2, the TEM image of AuNPs confirmed the uniformity of the spherical particles with a suitable size dispersion and also showed that the average size of the particles was around 15 nm.

To assess the stability of the developed nanostructure for *in vivo* animal study, the stability analysis of AuNPs-AFPA was assessed with *in vitro* serum incubation for a duration of 7 hr. Figure S2(A) shows that the AuNPs-AFPA remained completely unscathed following 7 hr serum incubation time.

Moreover, the stability analysis of AuNPs and AuNPs-AFPA was assessed by adding a high concentration of a salt solution. As a result, unlike AuNPs, AuNPs-AFPA was still stable despite the addition of increasing and high concentrations of the NaCl solution, indicating the protective effect of aptamers from the aggregation of AuNPs after the addition of the salt solution (Figure S2(B and C)).

***Drug loading***

**Figure 3:**
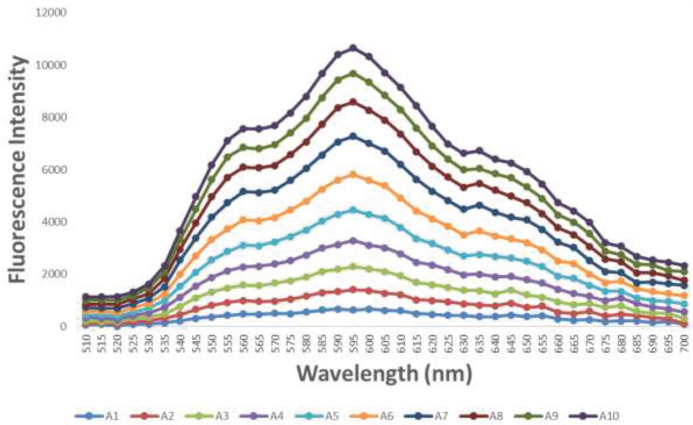
Fluorescence spectrum of Dox (1 µM) under growing concentrations of AuNPs-AFPA (top to bottom 0, 3.3, 8.25, 16.5, 33, 66, 99, 148.5, 198, and 300 nM)

Fluorometric analysis was performed for evaluation of Dox loading in AuNPs-AFPA structure, since upon Dox binding to DNA, the fluorescence of the drug is noticeably quenched (28). Therefore, fluorescence intensities of Dox (1 µM) were measured to escalate the loading rate of Dox into the AuNPs-AFPA structure under increasing concentrations of the final complex (0–300 nM). As displayed in Figure 3, when the quantity of the AuNPs-AFPA was 300 nM, the greatest fluorescence quenching of Dox occurred. Thus, the optimum mole ratio of Dox: AuNPs-AFPA was 3.33:1 and this ratio was used for the next experiments.

***In vitro pH-dependent Dox release***

**Figure 4:**
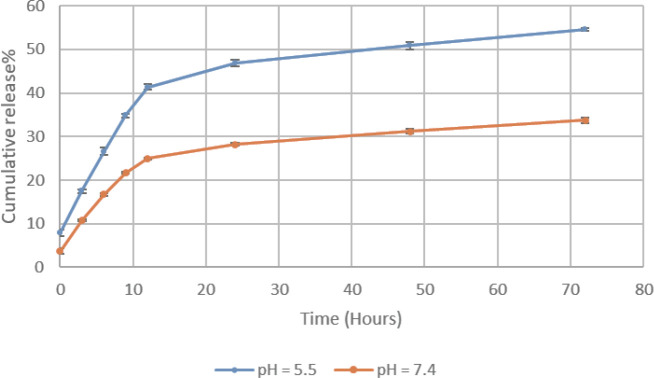
Release profile of Dox from AuNPs-AFPA at 37 °C in citrate buffer with pH = 5.5 (high) and phosphate buffer with pH = 4.7 (low) Data are means±SD (n = 3)

The release kinetic of Dox from the AuNPs-AFPA-Dox was considered at two various conditions (acidic condition with pH 5.5 and neutral condition with pH 7.4). As displayed in Figure 4, the release of Dox from AuNPs-AFPA-Dox within 72 hr was dependent on pH. In neutral conditions, approximately 34% of the Dox was released from AuNPs-AFPA-Dox over 72 hr, while in acidic conditions more release of Dox was observed from the structure at the same time (around 55%).

***In vitro*** **cell internalization evaluation**

**Figure 5:**
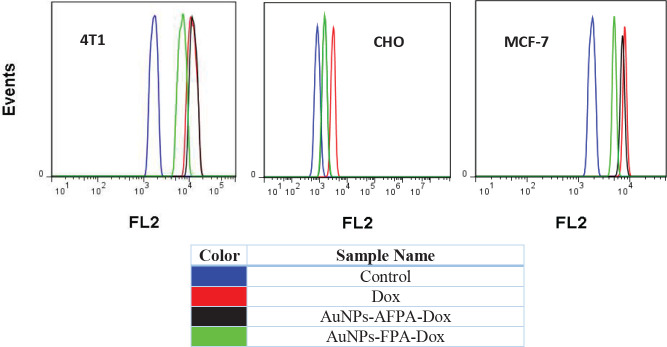
Flow cytometry histogram of CHO, MCF-7, and 4T1 cells after 3 hr incubation with AuNPs-AFPA-Dox, AuNPs-FPA-Dox, free Dox, and nontreated cells

**The c**ell internalization method was performed using flow cytometry analysis and a fluorescence microscope. The fluorescence FL2 histograms of MCF-7, CHO, and 4T1 cells following 3 hr incubation with AuNPs-AFPA-Dox, AuNPs FPA-Dox, and Dox have been displayed in Figure 5. 

**Figure 6:**
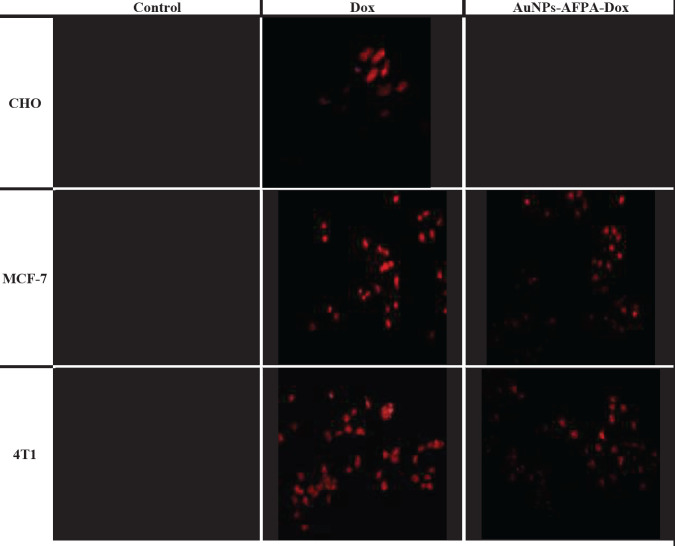
Fluorescence images of CHO, MCF-7, and 4T1 cells after 3 hr incubation with AuNPs-AFPA-Dox and free Dox

The cellular internalization images of Dox were taken after 3 hr incubation of CHO, MCF-7, and 4T1 cells with AuNPs-AFPA-Dox and Dox to further evaluate the cellular internalization of AuNPs-AFPA-Dox. Fluorescence images indicated that high concentrations of Dox were transferred into 4T1 and MCF-7 cells (nucleolin^+^, target) after treating with AuNPs-AFPA-Dox but in the CHO cell line (nucleolin^-^, nontarget), AuNPs-AFPA-Dox did not have fluorescence emission (Figure 6). However, the strong fluorescence emission of Dox in CHO, 4T1, and MCF-7 cells after 3 hr incubation of free Dox showed well internalization of free Dox into all three cell lines. The results were consistent with the data gained by flow cytometry analysis.

***In vitro cell viability assessment***

**Figure 7:**
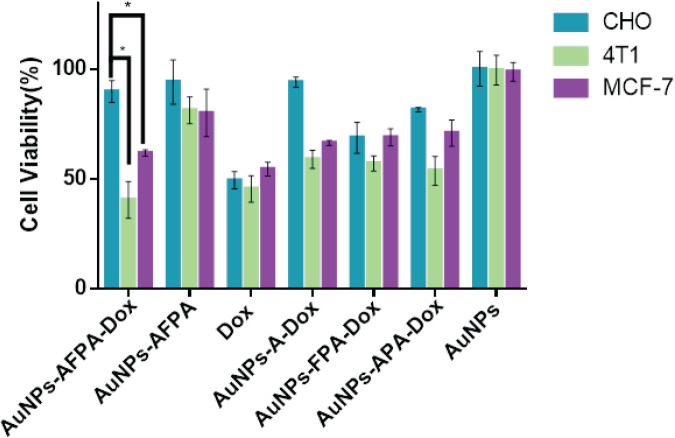
Effect of different groups on the viability ratio of control cells (CHO) and target cells (4T1 and MCF7)

MTT assay was performed to evaluate the toxicity of AuNPs-AFPA-Dox, AuNPs-AFPA, AuNPs-FPA-Dox, AuNPs-A-Dox, AuNPs-APA-Dox, AuNPs, and Dox on 4T1 (nucleolin+), MCF-7 and CHO (nucleolin^-^) cell lines (Figure 7). Cell viabilities for MCF-7 cells after treatments with AuNPs-AFPA-Dox, AuNPs-AFPA, AuNPs-FPA-Dox, AuNPs-A-Dox, AuNPs-APA-Dox, AuNPs and Dox were 61.9 ± 1.5%, 80.1 ± 5.3%, 69 ± 3.9%, 66.5 ± 1.1%, 70.9 ± 5.9%, 67.6 ± 4.7% and 54.8 ± 3.1%, for 4T1 cells were 40.5 ± 8.4%, 81.3 ± 6.1%, 57.2 ± 3.5%, 58.9 ± 2.5%, 53.7 ± 5.9%, 99.6 ± 6.8% and 45.5 ± 6.1% and for CHO cells were 89.9 ± 5.1%, 94.2 ± 2.5%, 65.4 ± 7.3%, 94.1 ± 2.3%, 81.6 ± 1.1%, 101.2 ± 4.7% and 49.5 ± 4.4%, respectively.

***In vivo evaluation of AuNPs-AFPA-Dox***

**Figure 8:**
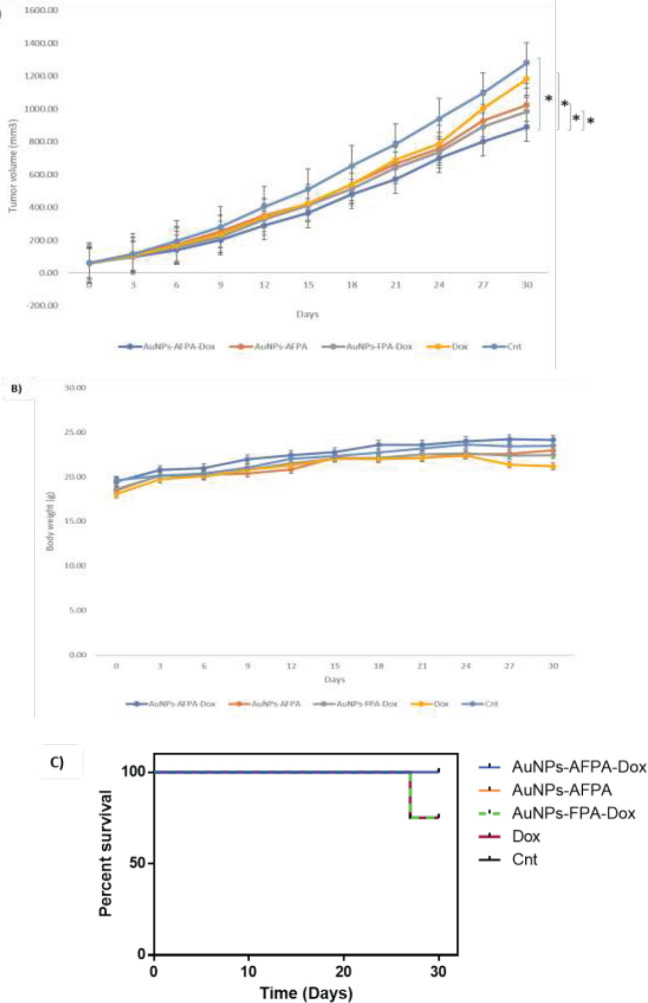
(A) Comparison of *in vivo* anticancer effectiveness after intravenous single-dose injections of AuNPs-AFPA-Dox, AuNPs-AFPA, AuNPs-FPA-Dox, Dox, and PBS via the tail vein in mice bearing 4T1 cells for 30 days (n = 4). (B) Comparison of weight changes in mice bearing 4T1 cells after single-dose injection of AuNPs-AFPA-Dox, AuNPs-AFPA, AuNPs-FPA-Dox, Dox, and PBS for 30 days (n = 4). (C) Kaplan-Meyer survival curve of 4T1 tumor model in mice treated with single intravenous doses of AuNPs-AFPA-Dox, AuNPs-AFPA, AuNPs-FPA-Dox, Dox, and PBS for 30 days

**For** *in vivo* evaluation of the therapeutic performance of AuNPs-AFPA-Dox, female mice bearing subcutaneous 4T1 tumors were divided into five experimental groups to be treated with AuNPs-AFPA-Dox, AuNPs-AFPA, AuNPs-FPA-Dox, free Dox, and PBS as a control group. Based on Figure 8A, the tumor volumes for AuNPs-AFPA-Dox, AuNPs-AFPA, AuNPs-FPA-Dox, free Dox, and PBS treated groups were 889.4 ± 21 mm^3^, 1024.7 ± 31 mm^3^, 984.6 ± 22 mm^3^, 1182.1 ± 28 mm^3^ and 1281 ± 21 mm^3^, respectively (after 30 days). As shown in Figure 8B, just the Dox-treated group showed noticeable body weight loss after 30 days. Moreover, Figure 8C indicates the survival rate of 4T1-tumor-bearing mice treated with AuNPs-AFPA-Dox, AuNPs-AFPA, AuNPs-FPA-Dox, free Dox, and PBS. After 30 days, no death was observed in the group treated with AuNPs-AFPA-Dox but some mortality was reported in the groups that received AuNPs-FPA-Dox and free Dox.

***Ex vivo biodistribution***

**Figure 9:**
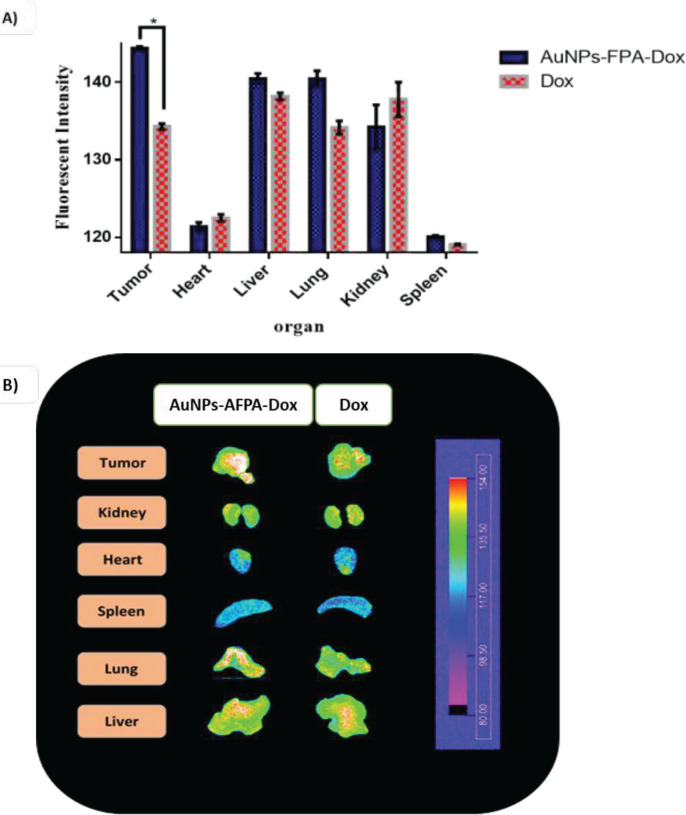
(A) ROI analysis and (B) *ex vivo *fluorescence images, 6 hr after injection of targeted formulation and Dox into mice bearing 4T1 tumor cells

The *ex vivo* fluorescence images were taken after 6 hr post intravenous injection of a single dose of AuNPs-AFPA-Dox and free Dox in female 4T1 tumor-bearing mice to further investigate the biological distribution of the AuNPs-AFPA-Dox *in vivo* and its accumulation in the tumor. Fluorescence images were taken from major organs including tumor, liver, heart, kidney, lung, and spleen tissues, and region of interest (ROI) for all tissues was analyzed applying KODAK Molecular Imaging software 5.0 (Figure 9A). According to Figure 9B, 6 hr post-injection of AuNPs-AFPA-Dox, the highest accumulation of fluorescence intensity was in tumor tissue.

## Discussion

Nowadays, one of the new strategies in cancer treatment is using aptamers as potential targeting agents for targeted drug delivery (38, 39). Targeted delivery of anticancer drugs using nanostructure carriers and aptamers can grant unique features for reducing the adverse effects of chemotherapeutic agents, improving their therapeutic effects (40, 41).

In the present study, a targeted treatment platform containing AuNPs as a carrier, FOXM1 Apt, AS1411, and ATP Apt was developed to treat breast cancer by co-delivery of Dox as a chemotherapy drug and FOXM1 Apt as a therapeutic aptamer to cancerous cells. The designed delivery system not only could be more effective and efficient to treat cancer but also had fewer side effects of Dox especially for nontarget cells in comparison with free Dox. Furthermore, the developed targeting platform has many positive points such as simple and cheap preparation of complex and high serum stability. Therefore, it can be concluded that the present targeting platform is a suitable carrier for Dox delivery.

FOXM1 Apt is a potent therapeutic agent via its capacity to efficiently hinder cancer cell proliferation by its attachment to FOXM1 protein and suppression of transcriptional activities of this protein. However, FOXM1 Apt cannot enter tumor cells alone because there is no specific ligand for this aptamer on the surface of the cell membrane and the aptamer is repelled through the negative charge of the cell membrane. In this study, to internalize FOXM1 Apt to cancer cells, it was bound to the AuNPs-AFPA-Dox owning AS1411 as a targeting agent. AS1411 was used to deliver all connected items into tumor cells perfectly. Furthermore, to improve the performance of this targeted drug delivery system, protect it against digestion by metabolic enzymes, and enhance its half-life, ATP apt was modified by PEGylation. As a result, this targeting structure is effective for transferring therapeutic agents into breast cancer cells.

The large surface area and high bioavailability of gold nanoparticles (AuNPs) have made them a suitable carrier for targeted drug delivery (42, 43). Using aptamers as targeting agents alongside AuNPs solved the inability of AuNPs to identify target cells alone (44-46). AuNPs are aggregated in the presence of a high concentration of salt solution. To protect AuNPs against salt-induced aggregation, conjugation with aptamers was performed (Figure S2 (B and C)). Also, AuNPs can protect aptamers against enzymatic degradation in the body (47, 48). Therefore, concomitant use of aptamer and AuNPs can be a valuable achievement for targeted drug delivery of chemotherapy drugs.

Drug loading into the AuNPs-AFPA was investigated by measuring the quenching property of Dox fluorescence after their incubation. Dox is intercalated among DNA base pairs of DNA structure in AuNPs-AFPA because Dox is preferentially loaded in the double-stranded regions. After incubating 300 nM AuNPs-AFPA with 1 µM Dox, Dox fluorescence intensity decreased (Figure 3), verifying the formation of AuNPs-AFPA-Dox with 1:3.33 mole ratio of AuNPs-AFPA:Dox. By comparing the present structure with various DNA nanostructures that have been used for targeted delivery of chemotherapeutic agents using aptamers as both carriers and targeting agents such as polyvalent system (mole ratio of complex to epirubicin was 1:2) (49) and Aptamer-Dox physical conjugate (mole ratio of Dox to aptamer was 1:1.2) (50), it can be concluded that AuNPs-AFPA had more vacancy to load drugs.

Figure 4 indicates the profile of Dox release from the AuNPs-AFPA-Dox in different pH within 72 hr. In pH 5.5 which simulates the conditions inside lysosomes, endosomes, and tumor tissues (51), there was more and rapid Dox release from the complex (around 55%) during 72 hr. In contrast, in pH 7.4 which simulates blood condition, Dox had much less release from the complex under the same conditions (nearly 34%). This pH-depending release of the drug can be related to protonation of the -NH_2_ group of Dox, reduction of the hydrophobic interaction between the aromatic ring of Dox and the bases of AuNPs-AFPA, and also enhancement of Dox solubility in acidic pH (51, 52). Therefore, the pattern of Dox release of AuNPs-AFPA-Dox under the acidic pH condition indicates the reduction of Dox side effects and improvement of its anticancer efficacy.

As shown in Figure 5, flow cytometry analysis exhibited significantly fewer fluorescence signals of CHO cells (nontarget) incubated with AuNPs-AFPA-Dox in comparison with Dox treatment (*P*<0.05), indicating less entrance into CHO cells treated with AuNPs-AFPA-Dox due to the lack of the target site of AS1411, called nucleolin, on the cell membrane of these cells. Moreover, the fluorescence intensity of 4T1 and MCF-7 cells (target) treated with AuNPs-AFPA-Dox raised as well as Dox treatment for these cells. Compared to Dox, AuNPs-AFPA-Dox was efficiently internalized into MCF-7 and 4T1 cells via AS1411-nucleolin interaction because these cells overexpress nucleolin on their surfaces. All three mentioned cell lines treated with AuNPs-FPA-Dox had fewer fluorescence signals compared to Dox treatment due to the lack of presence of AS1411 as a targeting agent. As a result, it can be verified that AuNPs-AFPA-Dox benefited from effective differentiation between nontarget and target cells.

The images of the fluorescence microscope further proved high internalization of AuNPs-AFPA-Dox into 4T1 and MCF-7 cells (target) unlike CHO cells (nontarget), confirming obtained internalization data from flow cytometry analysis (Figure 6). In contrast, internalization of Dox alone was high in all mentioned target and nontarget cell lines via its passive diffusion.

As shown in Figure 7, the results of the MTT assay substantiated the obtained previous internalization data. AuNPs-AFPA-Dox caused cell death almost like Dox in MCF-7 and 4T1 cells via AS1411-nucleolin interaction in these target cells. In contrast, in the CHO cells owing to the deficit of expression of nucleolin on the membrane of these cells, AuNPs-AFPA-Dox had negligible cytotoxicity compared to Dox alone (*P*<0.05). These results could be related to the presence of FOXM1 Apt and its function which inhibited FOXM1 protein activity and also the presence of AS1411 to detect nucleolin on the surface of target cells. AuNPs-AFPA was much less toxic in 4T1 and MCF-7 cells compared to AuNPs-AFPA-Dox due to the lack of presence of Dox. Also, AuNPs-FPA-Dox had almost the same toxicity in all three cell lines due to the lack of the presence of AS1411 as a targeting agent, showing the significant role of AS1411 for high entry of complex into target cells against nontarget cells. Furthermore, AuNPs-APA-Dox caused less mortality in MCF-7 and 4T1 cells than free Dox, due to the lack of presence of FOXM1 Apt and its therapeutic. AuNPs in none of the three cell lines caused cell death, indicating the non-toxicity of AuNPs alone. The AS1411 aptamer present in our complex enables it to function as a targeted drug delivery system, utilizing micropinocytosis, an active transport mechanism. This method is more effective than passive diffusion which the free drug relies on for cell entry.

To assess *in vivo* anti-tumor efficacy of AuNPs-AFPA-Dox, it was analyzed in BALB/c mice bearing 4T1 tumors. Based on the results obtained from the tumor growth assessment (Figure 8A), the AuNPs-AFPA-Dox group was able to remarkably suppress the tumor growth rate in comparison with AuNPs-AFPA, AuNPs-FPA-Dox, DOX, and PBS groups (*P*<0.05), confirming the high internalization and anti-tumor function of the present targeting delivery system (AuNPs-AFPA-Dox) in tumor cells. The AuNPs-FPA-Dox group showed less suppression of tumor growth than the AuNPs-AFPA-Dox group due to the lack of AS1411, indicating the necessity of the attendance of AS1411, as a targeting substance, in the complex for efficient cancer treatment. Also, the AuNPs-AFPA group had reduced tumor growth rate compared with the AuNPs-AFPA-Dox group due to the lack of Dox, displaying the importance of combination therapy (co-delivery of Dox and FOXM1 Apt) in the treatment of breast cancer. According to the survival chart of mice (Figure 8C), in the Dox group due to drug toxicity and in the AuNPs-FPA-Dox group owing to the presence of the Dox and the absence of the AS1411 as a targeting agent, the survival rate of mice was reduced (75%) compared to the other groups. In the AuNPs-AFPA-Dox group, all mice were alive and its survival rate was significantly higher than the Dox and AuNPs-FPA-Dox groups over 30 days (*P*<0.05), attributing to the lower side effects and the high efficiency of the complex. According to Figure 8B, AuNPs-AFPA-Dox, AuNPs-AFPA, and AuNPs-FPA-Dox groups did not show weight loss in mice. While in the Dox group, the weight of mice reduced (especially in recent days) due to the toxicity of the chemotherapy drug, demonstrating the mentioned *in vivo* results.

In the MTT assay, it was observed that AuNPs-AFPA exhibited lower toxicity than the free drug. It should be noted that while passive diffusion allows for entry of the free drug into all types of cells, in the MTT test only one type of cell was utilized and therefore all available free drugs were internalized into that cell line. AuNPs-AFPA as a target drug delivery system, in both *in vitro *and* in vivo* tests was internalized into target cells just by active transport and had almost the same toxicity in both tests. So, the toxicity of the free drug was more than AuNPs-AFPA *in vitro*. However, during *in vivo* testing, the free drug efficacy decreased and it exhibited reduced levels of toxicity when compared with AuNPs-AFPA.

As reported by the results of *ex vivo* imaging (Figure 9), 6 hr post-injection, the accumulation of AuNPs-AFPA-Dox in tumor tissue was significantly higher than Dox alone (*P*<0.05). This is due to the presence of AS1411 and its binding to nucleolin that is overexpressed on the membrane of different tumor cells, particularly in breast cancer. According to this binding, the survival time of the targeted formulations increased at the tumor site because of the delayed elimination of the formulation from the tumor tissue (53). As we know, one of the restricting parameters in using Dox in cancer treatment is its heart complication (54). Figure 9 showed that in cardiac tissue, the mean fluorescence intensity for mice treated with AuNPs-AFPA-Dox was less than for mice treated with Dox alone, verifying the lower toxicity of the complex due to its targeting function. Thus, these results demonstrate both the safety and efficacy of the designed AuNPs-AFPA-Dox for the treatment of breast cancer.

Dox: Doxorubicin, AuNPs-AFPA-Dox: The Dox-DNA nanostructure including AuNPs, AS1411, FOXM1 aptamer, PEGylated ATP aptamer and Dox.

PA: PEGylated ATP aptamer, FPA: FOXM1 aptamer-PEGylated ATP aptamer conjugate, AuNPs: The gold nanoparticles, AuNPs-AFPA: The DNA nanostructure including AuNPs, AS1411, FOXM1 aptamer, PEGylated ATP aptamer

TEM: Transmission electron microscopy, AuNPs: The gold nanoparticles

Dox: Doxorubicin, AuNPs-AFPA: The DNA nanostructure including AuNPs, AS1411, FOXM1 aptamer, PEGylated ATP aptamer

Dox: Doxorubicin, AuNPs-AFPA: The DNA nanostructure including AuNPs, AS1411, FOXM1 aptamer, PEGylated ATP aptamer

It should be mentioned that in CHO cells, the black curve had overlap with the green curve

Dox: Doxorubicin, AuNPs-AFPA-Dox: The Dox-DNA nanostructure including AuNPs, AS1411, FOXM1 aptamer, PEGylated ATP aptamer and Dox, AuNPs-FPA-Dox: The Dox-DNA nanostructure including AuNPs, FOXM1 aptamer, PEGylated ATP aptamer and Dox.

Dox: Doxorubicin, AuNPs-AFPA-Dox: The Dox-DNA nanostructure including AuNPs, AS1411, FOXM1 aptamer, PEGylated ATP aptamer and Dox.

Cells were treated for 3 hr. The cell viability was investigated after 72 hr using an MTT assay. Data are means±SD (n= 5).

Dox: Doxorubicin, AuNPs-AFPA-Dox: The Dox-DNA nanostructure including AuNPs, AS1411, FOXM1 aptamer, PEGylated ATP Aptamer and Dox, AuNPs-AFPA: The DNA nanostructure including AuNPs, AS1411, FOXM1 aptamer, PEGylated ATP aptamer, AuNPs-A-Dox: The Dox-DNA nanostructure including AuNPs, AS1411 and Dox, AuNPs-FPA-Dox: The Dox-DNA nanostructure including AuNPs, FOXM1 aptamer, PEGylated ATP aptamer and Dox, AuNPs-APA-Dox: The Dox-DNA nanostructure including AuNPs, FOXM1 aptamer, PEGylated ATP aptamer and Dox, AuNPs: Gold nanoparticles.

Data are means±SD (n= 4).

Dox: Doxorubicin, AuNPs-AFPA-Dox: The Dox-DNA nanostructure including AuNPs, AS1411, FOXM1 aptamer, PEGylated ATP aptamer and Dox, AuNPs-AFPA: The DNA nanostructure including AuNPs, AS1411, FOXM1 aptamer, PEGylated ATP aptamer, AuNPs-FPA-Dox: The Dox-DNA nanostructure including AuNPs, FOXM1 aptamer, PEGylated ATP aptamer.

Dox: Doxorubicin, AuNPs-AFPA-Dox: The Dox-DNA nanostructure including AuNPs, AS1411, FOXM1 aptamer, PEGylated ATP aptamer and Dox

## Conclusion

In summary, this study aimed to prepare a novel targeted delivery system using AuNPs as a carrier, AS1411 as a detector aptamer, ATP Apt (containing FOXM1 Apt complementary motif) to targeted co-delivery of FOXM1 Apt as a therapeutic molecule, and Dox to breast cancer cells. The therapeutic effectiveness of the prepared system was evaluated *in vitro* and *in vivo*. The combination therapy by Dox and FOXM1 Apt rendered augmentation in the antitumor efficacy index.

The results displayed that the presented targeted system (AuNPs-AFPA-Dox) due to the presence of AS1411, which increased the entry of therapeutic agents to target cancer cells (MCF7 and 4T1) by detecting nucleolin on the surface of these cells, could differentiate between target and nontarget cells (CHO), unlike free Dox. Moreover, according to MTT results, AuNPs-AFPA-Dox was effectively internalized into target cells (4T1 and MCF-7) unlike nontarget cells (CHO), and reduced their cell viability. The targeted formulation had other unique advantages including high drug loading, high stability, high specificity for target cells, easy design and production, uniform particle size, and pH-dependent drug release. The *in vivo *results showed the tumor growth inhibition property of AuNPs-AFPA-Dox that resulted in decreased side effects of Dox in comparison with free chemotherapy drug (Dox) administration. Besides, the *ex vivo* analysis exhibited that the toxicity of AuNPs-AFPA-Dox in the heart, as a non-cancerous and healthy tissue, was reduced.

Finally, it can be concluded that the designed targeted system can be used potentially as an efficient and safe strategy for the treatment of breast cancer which was easier to prepare and synthesize than previous studies. Also, due to the presence of FOXM1 Apt, the amount of required chemotherapy drug was reduced which significantly decreased the side effects of chemotherapy drugs. These results make AuNPs-AFPA-Dox an ideal targeted delivery strategy for efficient cancer therapy by its promising potential.

## Authors’ Contributions

A A helped investigate and write the original draft. SM T provided conceptualization, project administration, funding acquisition, writing, reviewing, and editing. K A provided supervision, conceptualization, writing, reviewing, and editing. M R contributed to writing, review, editing, and methodology. M A contributed to writing, review, editing, formal analysis, and validation. H R and E Y helped investigate, write, review, and edit. 

## Conflicts of Interest

There are no conflicts of interest in this article.
